# Clinical Nomogram for Predicting the Prognosis of Patients With Pulmonary Venous Obstruction After Total Anomalous Pulmonary Venous Connection Repair

**DOI:** 10.3389/fcvm.2022.733253

**Published:** 2022-02-16

**Authors:** Ling Chen, Zhihuang Qiu, Fan Xu, Xingfeng Chen, Liangwan Chen

**Affiliations:** ^1^Department of Cardiac Surgery, Fujian Medical University Union Hospital, Fuzhou, China; ^2^Fujian Key Laboratory of Cardio-Thoracic Surgery (Fujian Medical University), Fujian Province University, Fuzhou, China; ^3^Fujian Provincial Special Reserve Talents Laboratory, Fujian Province University, Fuzhou, China

**Keywords:** total anomalous pulmonary venous connection, nomogram, decision-making curve, postoperative pulmonary venous obstruction, preoperative pulmonary venous stenosis

## Abstract

**Background:**

The aim of this study was to establish a nomogram to quantify the risk of postoperative pulmonary venous obstruction (PVO) and to make a scientific decision through the decision curve.

**Methods:**

In total, 151 PVO patients with total anomalous pulmonary venous connection (TAPVC) repair in our hospital from December 2008 to December 2015 were involved in this study. A nomogram was generated based on the contribution weights of variables, which were found out by logistic analysis. The optimal clinical decision point was determined by the decision analysis and clinical impact curve, which could assess the net benefit between the nomogram and each independent risk factor for postoperative PVO.

**Result:**

Pulmonary venous obstruction with TAPVC repair was found to be positively and independently correlated with preoperative pulmonary hypertension, surgical methods, and preoperative pulmonary venous stenosis.

**Conclusion(s):**

The study introduced a novel model to aid in clinical decisions making for the patients with TAPVC individually, which may shed light on the evaluation of PVO risk.

## Introduction

The goal of surgical repair of total anomalous pulmonary venous connection (TAPVC) is to establish a channel connection between the pulmonary venous confluence and the left atrium. Postoperative pulmonary venous obstruction (PVO) associated with TAPVC is a serious postoperative complication of this procedure. The incidence of PVO is between 5 and 18% (1–4). Data on the prognosis of all patients born with TAPVC in the United Kingdom, Ireland, and Sweden over a period of 7 years (*n* = 422) were reported. Postoperative PVO was found to be an important risk factor for death, occurring in 71 (17.5%) of 406 patients who underwent repair of TAPVC.

Therefore, determining the risk factors of postoperative venous stenosis and quantifying the risk of its occurrence are essential for clinical decision-making and clinical care. However, the existing literature only demonstrates that preoperative venous stenosis is a risk factor for postoperative PVO in patients with TAPVC, which has a certain predictive value. But it has not carried out an objective quantitative assessment and risk prediction of its risks (5–7). Judging by subjective experience alone is not rigorous enough, lacks a reliable basis, and does not have a consistent threshold, which may lead to variability in clinical decision-making and inappropriate risk stratification.

By searching multiple databases, namely, PubMed, the nomogram prediction model has been widely and successfully used for prognosis prediction and survival analysis of various diseases (8–11). The risk is quantified by analyzing and considering all known clinical variables, thereby allowing individualized risk assessment and prognosis prediction for multiple diseases. At present, no relevant predictive model has been established at home and abroad to quantitatively evaluate the risk of postoperative PVO in patients with TAPVC. Therefore, this study intends to include all patients with TAPVC, determine the risk factors of postoperative PVO, establish a nomogram prediction model to quantify the risk of postoperative PVO, and provide an objective reference for clinical decision-making.

## Methods

A retrospective analysis of all patients who underwent TAPVC repair in our hospital from December 2008 to December 2015 was performed, and the enrollment of the required cases was completed within 1 year. Due to the retrospective nature of the study, informed consent was abandoned. Patients were identified by searching technical databases, such as surgical systems, imaging systems, and surgical systems, for “total anomalous pulmonary venous connection,” “total anomalous pulmonary venous return,” equivalent acronyms, and related international disease classification codes. All imaging modalities (echocardiogram, cardiac magnetic resonance, CT, and catheterization) were reviewed to confirm the anatomic diagnosis. Clinical information was extracted from the electronic medical record, and operative notes were reviewed.

### Study Definitions

#### Pulmonary Vein Stenosis and Obstruction

Because the definition of preoperative obstruction is very different in the existing literature, we defined the grade of preoperative obstruction to evaluate whether the increase of preoperative obstruction can better predict postoperative obstruction. The selection of threshold is based on the common values in the existing literature. Preoperative and postoperative transthoracic echocardiographic reports and intraoperative transesophageal echocardiography (TEE) reports were reviewed. The reported degree of stenosis in each pulmonary vein was graded from 0 to 3 (0 = no stenosis; 1 = mild stenosis with the mean pressure gradient <5 mm Hg; 2 = severe stenosis with a mean pressure gradient of more than 5 mm Hg; and 3 = occluded) based on echocardiographic measurement. The sum of these scores (four pulmonary veins) was defined as the PVS score (possible range, 0–12). Preoperative unilateral pulmonary vein stenosis was defined as the presence of stenosis in a confluence, vertical vein, or individual pulmonary veins. Preoperative bilateral pulmonary vein stenosis was defined as stenosis at either both confluences or both individual pulmonary veins (single or two per side) (12, 13).

#### Types of Postoperative Pulmonary Vein Stenosis

Based on the pathomorphological characteristics of the pulmonary vein, combined with preoperative imaging data and intraoperative anatomical findings, postoperative pulmonary vein stenosis can be divided into four types. Type I, anastomotic stenosis of the pulmonary vein: stenosis is caused by the fibrous scar hyperplasia at the junction of the pulmonary veins and the left atrial anastomosis, which can form “fish mouth” or “three-chamber heart”-like changes. The left and right branch pulmonary veins can be normal or narrow. Type II, initial stenosis of the pulmonary vein: the intimal hyperplasia where one side of pulmonary vein or one pulmonary vein enters the left atrium forms a narrow ring, the distal pulmonary veins are well developed. Type III, segmental stenosis of the pulmonary vein: segmental stenosis of the pulmonary vein was found in the proximal part of the left atrial junction, and the distal pulmonary vein was dilated or normal. Type IV, diffuse stenosis or occlusion of the pulmonary vein: one side of the pulmonary vein or one pulmonary vein is diffusely narrowed, or the proximal end is completely occluded. The distal pulmonary veins are diffusely narrowed or completely occluded in a cord-like shape (14).

#### Types of the Mixed TAPVC

The type of mixed TAPVC can be divided into three parts combined with the pulmonary vein arrangement of mixed TAPVC in the existing literature. Firstly, all pulmonary veins were drained into one confluence, and the confluence had two separate vertical veins; secondly, 1 + 3 bilateral asymmetrical connections with a solitary pulmonary vein and the other three pulmonary veins; and thirdly, 2 + 2 bilateral symmetrical connection with separate anomalous pulmonary veins from each lung (12).

### Statistical Analysis

The continuous variables were presented as mean ± SD or median with quartiles, and the categorical variables were presented as number and percentage. Differences in surgical technique between surgeons were assessed with the Chi-square test or Fisher's exact test. Kaplan-Meier analysis was performed to graphically display the time to postoperative obstruction and describe the survival rate of patients, and the Cox proportional hazard model was used to test the relationship between risk factors and death. Clinical risk factors for obstruction were identified using univariable logistic proportional hazards models. The same methods were used in two subgroups of patients: non-sutureless group and sutureless group. All the significant variables identified in the multivariable logistic regression analysis after identifying variables associated with obstruction in the univariable analyses were utilized to generate another logistic model and converted to a nomogram by library “rms” in R to predict the risk of PVO. The variable with the largest coefficient absolute value was set as a reference whose scale range was from 0 to 100. The performance of the nomogram was examined by the concordance index (c-index) and assessed by the calibration plot. The calibration plot was generated by 1,000 bootstrapped replications internally to illustrate the association between actual probability and the predicted probability. Clinical utility was estimated by decision curve analysis (DCA) and clinical impact curve, using the library “risk model decision analysis (rmda)” in R (23). DCA compared the net benefits of each prediction model at any threshold probability. Net benefit = (true positives/N) – (false positives/N) × (weighting factor). Weighting factor = Threshold probability/(1 – threshold probability). A two-tailed *p* <0.05 was considered statistically significant.

All statistical analyses were performed using SPSS21for mac (Chicago, IL, USA) and RStudio-1.2.1335 (http://www.r-project) with packages of Hmisc, ggplot2, survminer, rmda, and mass, etc.

## Results

### Characteristics and Baseline of the Participants

This is a retrospective study using patient samples obtained from those who 151 received their first TAPVC repair. The baseline data of 151 patients in the cohort are summarized in Table 1. The average age of the patients was 3.54 ± 4.42 months, and the average weight was 4.82 ± 1.65 kg. The cohort is predominantly male [n = 102 (67.5%)], i.e., 66 cases (43.7%) with supracardiac-type TAPVC, 28 cases (18.4%) with cardiac-type TAPVC, 3 cases (2.0%) suffered from intracardiac-type TAPVC, and 5 cases (3.3%) suffered from mixed-type TAPVC. In the female cohort [n = 49 (32.5%)] there are 34 cases (22.5%) with supracardiac-type TAPVC, 14 cases (9.3%) with cardiac-type TAPVC, and 1 cases (0.7%) suffered from intracardiac-type TAPVC. Preoperative pulmonary venous stenosis was associated with 38 cases (25.2%). Echocardiography or angiography showed that there were 18 cases (11.9%) with PVO before discharge. Postoperative complications included 10 cases of low cardiac output syndrome (6.6%), 3 cases of postoperative pulmonary hypertension (2.0%), 5 cases of postoperative pneumothorax (3.3%), 5 cases of postoperative pleural effusion (3.3%), and 8 cases of postoperative multiple organ failure (MOF) (5.3%). Ten patients (6.6%) died within 30 days in the hospital.

**Table 1:** Preoperative patient characteristics.

**Subgroup**	**Non-sutureless group (% or σ)**	**Sutureless group (% or σ)**	***P*** -value^*^
Overall	90 (59.6)	61 (40.4)	/
Age (month)	4.01 (5.04)	2.84 (3.22)	0.109
Weight (kg)	4.99 (1.77)	4.58 (1.44)	0.131
Sex			0.325
Male	58 (38.4)	44 (29.1)	/
Female	32 (21.2)	17 (11.3)	/
Types of TAPVC			0.285
Supracardiac	63 (41.7)	37 (24.5)	0.243
Cardiac	22 (14.7)	20 (13.2)	0.273
Infracardiac	2 (1.3)	2 (1.3)	0.694
Mixed	3 (2.0)	2 (1.3)	0.985
1 + 3	2 (1.3)	1 (0.6)	/
2 + 2	1 (0.7)	1 (0.7)	/
Other^a^	0 (0)	0 (0)	/
**Preoperative factor**	
Stenosis (preoperative)	21 (13.9)	16 (10.6)	0.806
Mild stenosis	9 (6.0)	8 (5.3)	0.555
Severe stenosis	4 (2.6)	3 (2.0)	0.893
Occluded	8 (5.3)	5 (3.3)	0.883
Pulmonary hypertension (preoperative)	69.44 (30.92)	61.20 (37.86)	0.144
**Pulmonary hypertension (degree)**	
None	5 (3.3)	6 (4.0)	0.349
Mild	22 (14.7)	10 (6.5)	0.291
Moderate	25 (16.5)	16 (10.6)	0.835
Severe	38 (25.2)	29 (19.2)	0.613
CPBT (min)	97.74 (40.63)	107.93 (45.52)	0.152
Aorta clamping (min)	56.63 (38.39)	53.03 (28.74)	0.534
Delayed sternal closure	0 (0)	0 (0)	/
Mechanical ventilation (h)	118.46 (160.01)	139.58 (142.38)	0.407
Surgery (During Neonatal period )	22 (14.7)	19 (12.6)	0.779
**Postoperative complications**	
LCOS (Low cardiac output syndrome)	6 (6.7)	4 (6.6)	0.979
Pulmonary hypertension	4 (4.4)	1 (1.6)	0.083
Pneumothorax	3 (3.4)	2 (3.3)	0.985
Pleural effusion	3 (3.4)	2 (3.3)	0.986
MOF (multiple organ failure)	7 (7.8)	1 (1.6)	0.063
Stenosis (postoperative)	20 (22.2)	3(5)	0.001
I	3 (3.4)	0 (0)	0.083
II	6 (6.6)	2 (3.3)	0.365
III	1 (1.1)	1 (1.7)	0.782
IV	3 (3.4)	0	0.083
I + II	4 (4.4)	0	0.096
II + III	2 (2.2)	0	0.158
II + IV	1 (1.1)	0	0.412
Postoperative hospitalized day	20.97 (15.69)	17.28 (8.13)	0.061
**Death rate**	
Total death	10 (11.1)	4 (6.6)	0.463
PVO	5 (5.6)	0	0.025
Other causes	5 (5.6)	4 (6.6)	0.801
Postoperative death (<30 days)	7 (7.8)	3 (4.9)	0.491

*CI, confidence interval; IQR, interquartile range; CPBT, Cardiopulmonary Bypass time*.

a *All pulmonary veins drained into one confluence, and the confluence had two separate vertical veins. Pulmonary vein arrangement: 1 + 3, bilateral asymmetrical connections with a solitary pulmonary vein and the other three pulmonary veins; 2 + 2, bilateral symmetrical connection with separate anomalous pulmonary veins from each lung. Total anomalous pulmonary venous drainage (TAPVD) type: 1, supracardiac; 2, cardiac; 3, infracardiac; 4, mix.Type of Stenosis ( postoperative): I, Anastomotic stenosis of pulmonary vein; II, Initial stenosis of pulmonary vein; III, Segmental stenosis of pulmonary vein; IV, Diffuse stenosis or occlusion of pulmonary vein*.

* *Chi-square test or Fisher's exact test*.

During the follow-up period, the mean follow-up time was 32.5 months. 23 patients include the patients before discharge were diagnosed with postoperative venous obstruction by echocardiography or angiography (15.2%). By the end of the follow-up period, 14 patients were died (9.3%), i.e., ten patients (6.6%) who died within 30 days in the hospital. The survival rate was 90.7%. Three patients were declared clinically dead due to severe multi-organ failure with TAPVC repair without any postoperative imaging examination and were considered to have reached a major outcome on the day of surgery. Eight patients (5.3%) with no postoperative venous obstruction died from other causes (Not defined as PV obstruction), and although it was impossible to determine the cause of death (e.g., sudden death at home) in all cases, we did not find any other possible causes of postoperative venous obstruction (Table 1).

### Survival Significance of Postoperative Pulmonary Venous Stenosis

**Figure 1:**
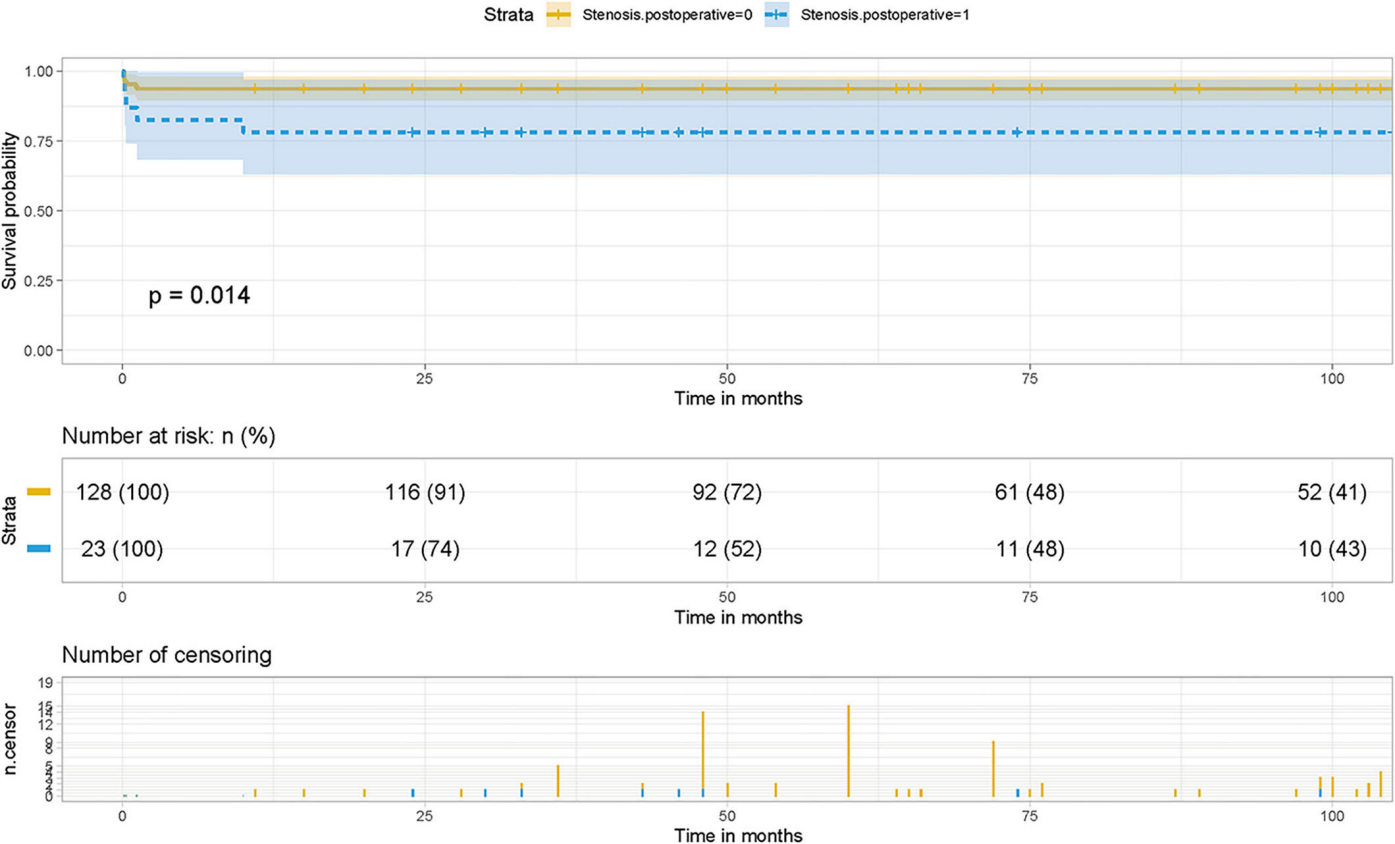
Effect of PVO on postoperative survival in patients with TAPVC and the cumulative risk (log-rank test). The x-axis and y-axis represent the postoperative follow-up time and survival probability, respectively [in yellow (Stenosis. postoperative = 0, negative pulmonary venous obstruction), the line represents the survival curve, and the width of the yellow bar represents the cumulative risk (the larger the width, the higher the cumulative risk). In blue (Stenosis. postoperative = 1, positive pulmonary venous obstruction), the line represents the survival curve, and the width of the blue bar represents the cumulative risk (the greater the width, the higher the cumulative risk)].

Kaplan-Meier survival curves and cumulative risk curves were established by COX analysis to assess the impact of postoperative PVO on patient survival. It is not difficult to find that postoperative PVO has a statistically significant impact on the long-term survival of patients with TAPVC repair (*p* = 0.00014), suggesting that with the extension of survival time, the cumulative risk of postoperative PVO increases, the survival probability of patients decreases, and the survival time is shortened (Figure 1).

### Contribution of the Factors to Postoperative PVO

We separately assessed whether each demographic or clinical risk factor was associated with PVO using univariable logistic proportional hazards models (Table 2). Preoperative pulmonary venous hypertension, preoperative pulmonary venous stenosis, and surgical methods are the risk factors for PVO with TAPVC repair. Furthermore, the results showed that severe stenosis or occlusion, moderate and severe pulmonary hypertension were the important risk factors of PVO. While gender (*p* = 0.823), age (*p* = 0.852), weight (*p* = 0.504), types of TAPVC (*p* = 0.198), Cardiopulmonary bypass time (extracorporeal circulation time) (*p* = 0.105), aorta clamping time (*p* = 0.605), and mechanical ventilation time (*p* = 0.286) were not risk factors for PVO. The previous risk factors were incorporated into the multivariate logistic risk model for further analysis. The results showed that preoperative pulmonary venous hypertension (*p* = 0.009), preoperative pulmonary vein stenosis (*p* = 0.001), and surgical methods (*p* = 0.003) were independently related to postoperative pulmonary vein stenosis (Tables 2, 3).

**Table 2:** Univariable Logistic proportional hazards models for the development of postoperative pulmonary venous obstruction.

**Subgroup**	***n*** **(%) or Median (IQR)**	**Hazard ratio (95 %CI)**	***P*** **-value**
Overall	151 (100)	/	/
Age (month)	2.0 (0.9-6.0)	3.54 (2.83-4.25)	0.852
Weight (kg)	4.5 (3.9-5.5)	4.82 (4.56-5.09)	0.504
Sex	/	0.68 (0.60-0.75)	0.823
Male	102 (67.5)	/	/
Female	49 (32.5)	/	/
Types of TAPVC	151 (100)	1.68 (1.52-1.84)	0.198
Supracardiac	100 (66.2)	0.66 (0.59-0.74)	0.191
Cardiac	42 (27.8)	0.03 (0.0006-0.05)	0.098
Infracardiac	4 (2.6)	0.28 (0.21-0.35)	0.588
Mixed	5 (3.3)	0.03 (0.004-0.06)	0.764
**Preoperative factor**			
Stenosis (preoperative)	38 (25.2)	0.25 (0.18-0.32)	0.000423
Mild stenosis	17 (11.3)	0.11 (0.06-0.116)	0.279
Severe stenosis	7 (4.6)	0.05 (0.01-0.08)	0.055
Occluded	13 (8.6)	0.09 (0.04-0.13)	0.000047
Pulmonary hypertension (preoperative)	61 (45-80)	66.58 (61.03-72.14)	0.000303
**Pulmonary hypertension (degree)**			
None	12 (8.0)	0.07 (0.03-0.11)	0.999
Mild	31 (20.4)	0.21 (0.14-0.27)	0.998
Moderate	41 (27.2)	0.27 (0.19-0.34)	0.027
Severe	67 (44.4)	0.45 (0.37-0.53)	0.000413
CPBT (min)	91 (67-135)	101.86 (94.98-108.75)	0.105
Aorta clamping (min)	48 (32-71)	55.18 (49.59-60.77)	0.605
Delayed sternal closure	0 (0)	/	/
Mechanical ventilation (h)	96 (57-144)	126.99 (102.39-151.59)	0.286
Surgery (during neonatal period)	41 (27.2)	0.71 (0.64-0.78)	0.519
Surgery (sutureless or Non-sutureless)	1 (0-1)	0.6 (0.52-0.68)	0.008

*CI, confidence interval; IQR, interquartile range; CPBT, Cardiopulmonary Bypass time*.

**Table 3:** Multivariable model for risk factors associated with the development of postoperative pulmonary venous obstruction.

**Subgroup**	**B**	**95%CI for OR**	***P*** **-Value**
Stenosis(preoperative)	0.873	1.458-3.932	0.001
Pulmonary hypertension(preoperative)	0.02	1.005-1.035	0.009
Surgery(Sutureless or Routine)	2.54	2.407-66.814	0.003

*B, regression coefficient; OR, odds ratio; CI, confidence interval*.

### Subgroup Analyses

Subgroup analysis was performed according to the operation method. No new risk factors were found in the non-sutureless group and preoperative pulmonary hypertension and preoperative pulmonary vein stenosis were still risk factors for PVO with TAPVC repair in the group. However, pulmonary hypertension was not a risk factor for PVO with TAPVC repair, except that pulmonary vein stenosis was still a risk factor for PVO with TAPVC repair in the sutureless group (Table 4).

**Table 4:** Subgroup analysis of patients with postoperative PVO.

**Subgroup**	**Non-sutureless group**			**Sutureless group**		
	***n*** **(%) or Median (IQR)**	**Hazard ratio (95 %CI)**	***P*** -value^*^	***n*** **(%) or median (IQR)**	**Hazard ratio (95 %CI)**	***P*** -value^*^
Overall	90 (100)	/	/	61 (100)	/	/
Age (month)	2 (0.9-6)	4.01 (3.00-5.07)	0.949	2 (0.9-4)	2.84 (2.02-3.66)	0.34
Weight (kg)	4.5(4–6)	4.99 (4.62-5.37)	0.575	4 (3.5-5.5)	4.58 (4.21-4.95)	0.501
Sex	/	0.64 (0.54-0.75)	0.557	/	0.72 (0.61-0.84)	0.829
Male	58 (64.4)	/	/	44 (72.1)	/	/
Female	32 (35.6)	/	/	17 (27.9)	/	/
Types of TAPVC	90 (100)	1.61 (1.41-1.81)	0.56	61 (100)	1.79 (1.53-2.05)	0.997
Supracardiac	63 (70)	0.70 (0.60-0.79)	0.581	37 (60.7)	0.61 (0.48-0.73)	0.998
Cardiac	22 (24.4)	0.22 (0.01-0.05)	0.273	20 (32.8)	0.32 (0.01-0.07)	0.89
Infracardiac	2 (2.2)	0.24 (0.15-0.34)	0.37	2 (3.3)	0.33 (0.21-0.45)	0.72
Mixed	3 (3.4)	0.03 (0.004-0.07)	0.642	2 (3.2)	0.03 (0.01-0.08)	0.93
1 + 3	2	/	/	1	/	/
2 + 2	1	/	/	1	/	/
Other^a^	0	/	/	0	/	/
**Preoperative factor**	
Stenosis (preoperative)	21 (23.3)	0.46 (0.26-0.65)	0.001	16 (26.2)	0.48 (0.24-0.71)	0.022
Mild stenosis	9	0.10 (0.04-0.16)	0.412	8	0.13 (0.04-0.22)	0.981
Severe stenosis	4	0.044 (0.001-0.087)	0.035	3	0.049 (0.001-0.105)	0.641
Occluded	8	0.089 (0.029-0.149)	0.002	5	0.082 (0.011-0.152)	0.008
Pulmonary hypertension (preoperative)	66 (50-83)	69.44 (62.97-75.92)	0.004	58 (48-89)	62.36 (52.32-72.40)	0.031
Pulmonary hypertension (degree)	2 (1-3)	2.09 (1.89-2.29)	/	2 (1-3)	2.11 (1.85-2.38)	/
None (0)	5	0.055 (0.007-0.104)	0.999	6	0.098 (0.022-0.175)	0.999
Mild (1)	22	0.23 (0.14-0.32)	0.798	10	0.16 (0.07-0.26)	0.999
Moderate (2)	25	0.28 (0.18-0.37)	0.03	16	0.26 (0.14-0.38)	0.999
Severe (3)	38	0.43 (0.33-0.54)	0.000269	29	0.48 (0.35-0.60)	0.786
CPBT (min)	89 (65-135)	97.74 (89.23-106.26)	0.319	92 (76-139.5)	107.93 (96.28-119.59)	0.282
Aorta clamping (min)	48 (35-71)	56.63 (48.59-64.67)	0.798	47 (30.5-70)	53.03 (45.67-60.39)	0.208
Delayed sternal closure	0 (0)	/	/	0 (0)	/	/
Mechanical ventilation (h)	72 (48-144)	118.46 (84.94-151.97)	0.394	116 (72-160)	139.58 (103.12-176.05)	0.888
Surgery (During Neonatal period)	22 (24.4)	0.70 (0.60-0.79)	0.272	19 (31.1)	0.72 (0.61-0.84)	0.998

*CI, confidence interval; IQR, interquartile range; CPBT, Cardiopulmonary Bypass time*.

a *All pulmonary veins drained into one confluence, and the confluence had two separate vertical veins. Pulmonary vein arrangement: 1 + 3, bilateral asymmetrical connections with a solitary pulmonary vein and the other three pulmonary veins; 2 + 2, bilateral symmetrical connection with separate anomalous pulmonary veins from each lung. Total anomalous pulmonary venous drainage (TAPVD) type: 1, supracardiac; 2, cardiac; 3, infracardiac; 4, mix. Type of Stenosis (prostoperative): I, Anastomotic stenosis of pulmonary vein; II, Initial stenosis of pulmonary vein; III, Segmental stenosis of pulmonary vein; IV, Diffuse stenosis or occlusion of pulmonary vein*.

* *Univariable Logistic analyze*.

### Establishment of PVO Score by Nomogram

**Figure 2:**
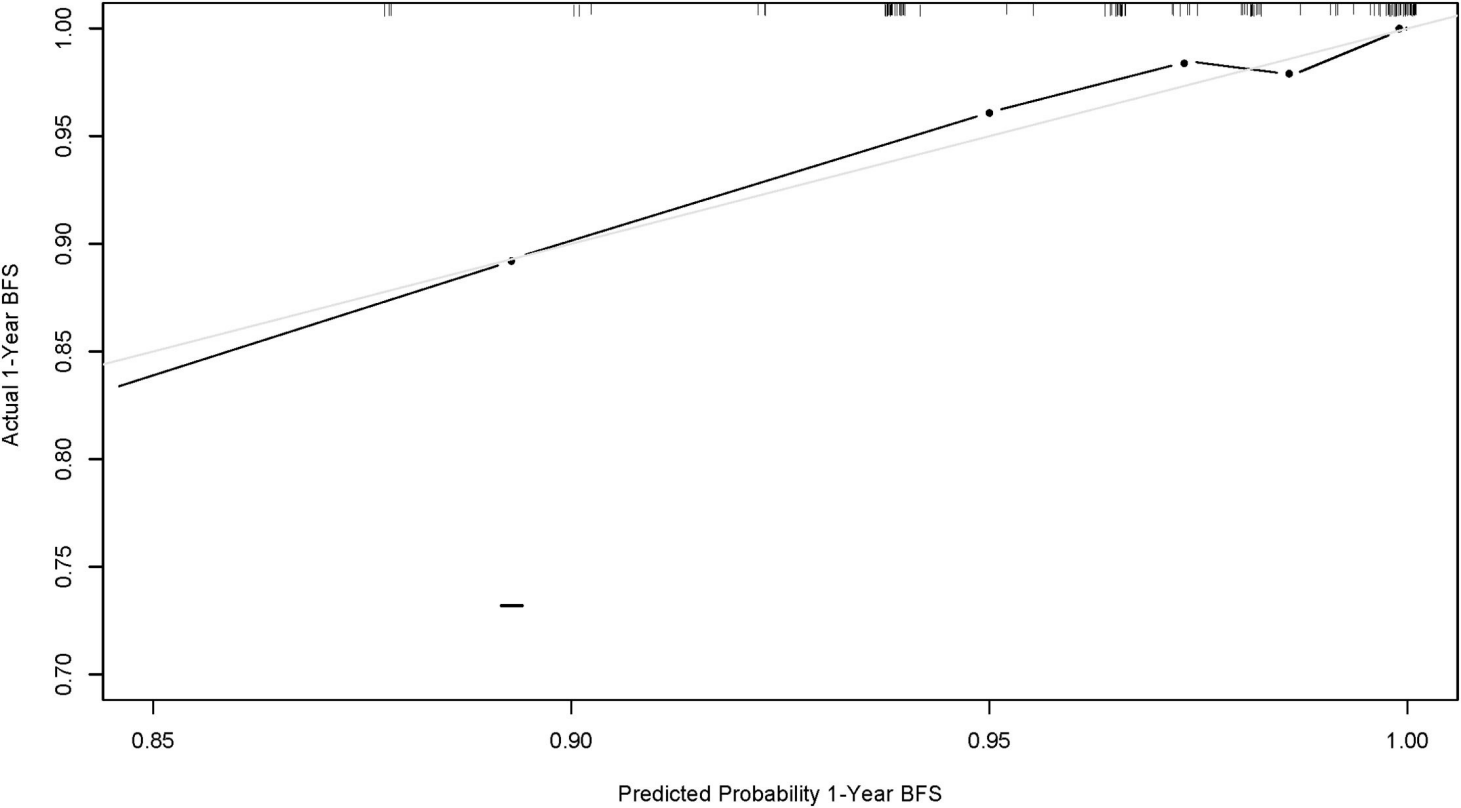
Calibration curve of nomogram model (1-year overall survival of this population). The x-axis and y-axis represent the predicted 1-year survival probability and the actual survival probability of the nomogram model, respectively.

**Figure 3:**
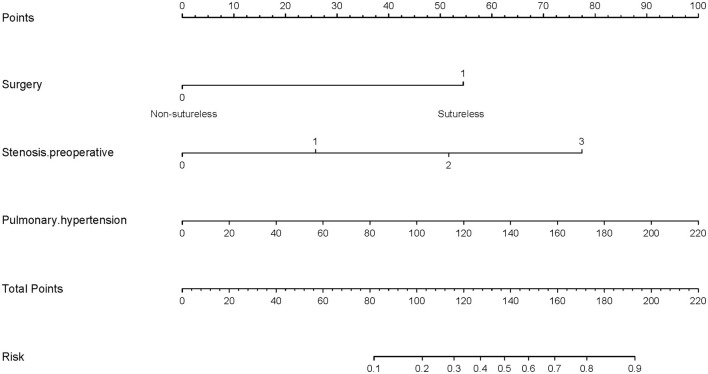
Generation of a nomogram. A nomogram for prediction of PVO risk for patients with TAPVC. The percentage of preoperative pulmonary hypertension, the variable which had the largest coefficient absolute value was set as reference whose scale range was from 0 to 100. Instructions for use of the nomogram: first, obtain the points of each characteristic of the patients by drawing a vertical line from each variable to the scale above (i.e., pulmonary hypertension, Surgery, and Stenosis. preoperative). Total points would be summed. The total score could be converted to the PVO risk by the alignment of the “total point” bar and the “probability of failure” axis, or vice versa.

**Figure 4:**
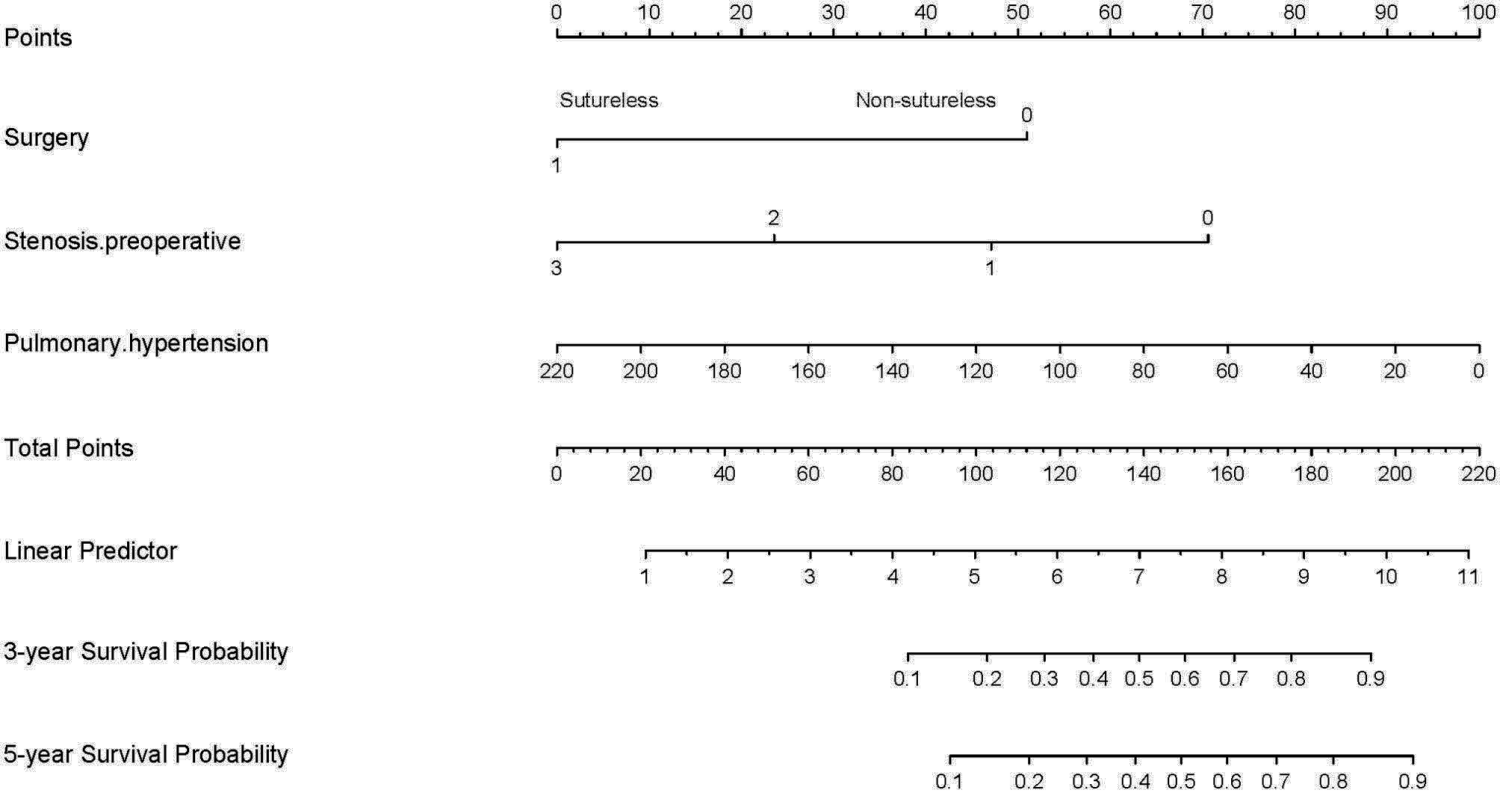
Generation of a nomogram. A nomogram for prediction of PVO 3-year and 5-year overall survival rates for patients with TAPVC. The percentage of preoperative pulmonary hypertension, the variable which had the largest coefficient absolute value was set as reference whose scale range was from 0 to 100. Instructions for use of the nomogram: first, obtain the points of each characteristic of the patients by drawing a vertical line from each variable to the scale above (i.e., pulmonary hypertension, Surgery, and Stenosis. preoperative). Total points would be summed. The total score could be converted to the PVO 3-year and 5-year overall survival rates by the alignment of the “total point” bar and the “probability of failure” axis, or vice versa.

To visualize the logistic proportional hazards models results, the significantly different variables in Table 2, preoperative pulmonary hypertension, preoperative pulmonary venous stenosis, and surgical method, were imported into R library “rms” for analysis. A nomogram is generated based on the contribution weights of variables. The prediction of postoperative individual risk of PVO and postoperative 3–5 years survival probability of patients who underwent TAPVC repair were determined by the total score value of each individual risk factor corresponding to the top of the scale. The nomogram showed good accuracy in predicting the possibility of postoperative PVO (Figure 2), with a C-index of 0.805 (95% CI: 0.732–0.878). The calibration diagram shows that the nomogram model has been correctly calibrated, with an average absolute error of 0.084 (Figures 3, 4).

### Determination of Decision Point for Maximum Clinical Benefit

**Figure 5:**
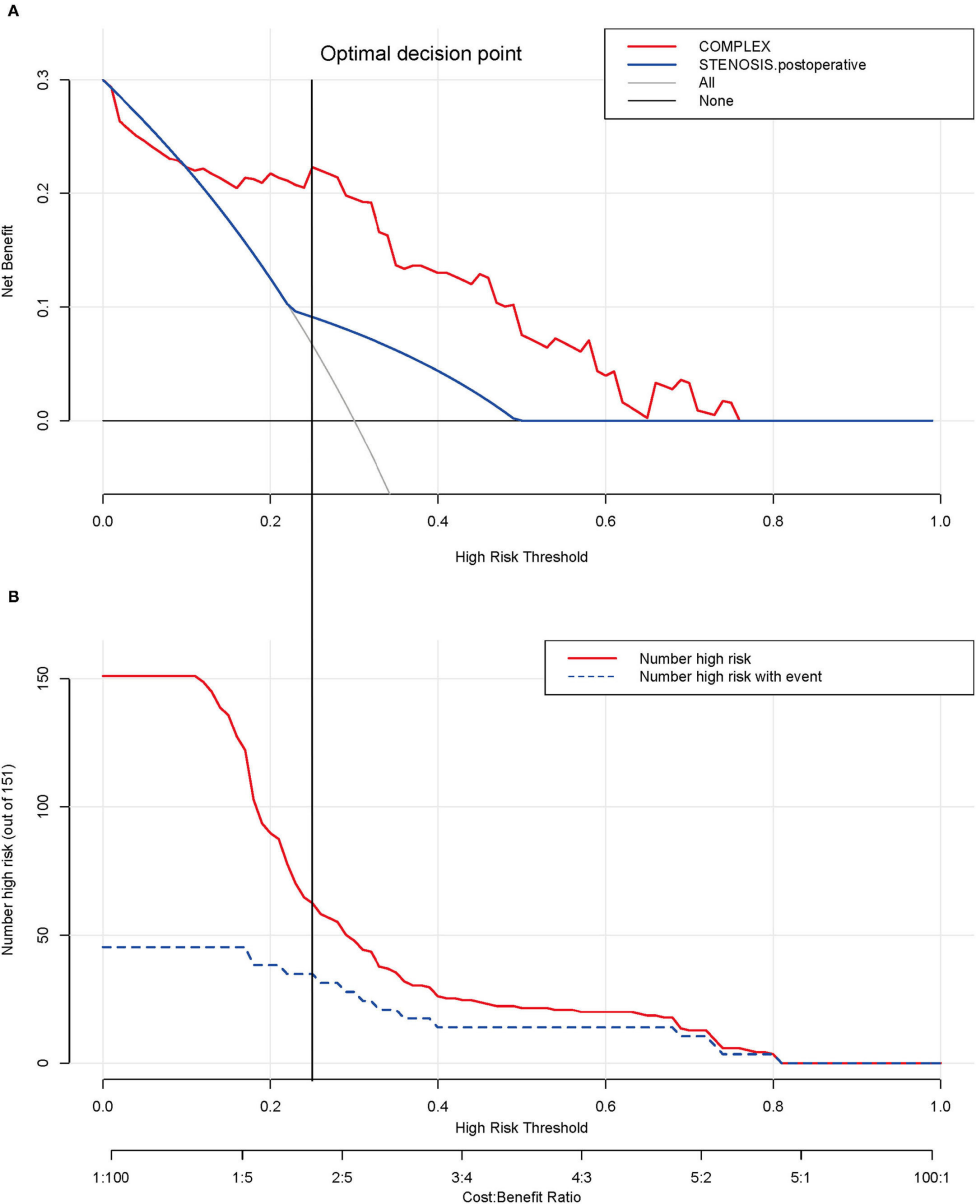
Determination of decision point *via* decision curve analysis (DCA) and clinical impact curve. **(A)** Decision curve for the prediction model. The decision curve analysis graphically shows the clinical usefulness of the nomogram based on a continuum of potential thresholds for PVO (x-axis) and the net benefit of using the nomogram to stratify patients (y-axis). Net benefit curves are plotted across probability thresholds for 4 options: “all” assume all patients have PVO, “none” assume no patients have PVO. Net benefit = (true positives/N) – (false positives/N) × (weighting factor). Weighting factor = threshold probability/(1 – threshold probability). **(B)** Clinical impact curve for nomogram. The red line shows the total number who would be deemed as high risk of PVO for each risk threshold. The blue line shows how many of those would be true positive (implantation failure). The vertical brown lines across the **(A)** and **(B)** showed the alignment of the DCA and the clinical impact curve to achieve the balance between the higher net benefits and lower false-positive rates, in which the threshold is set at 0.24, falling at 108 points in total at Figure 2

Decision analysis and the clinical impact curve were used to determine the optimal decision point. First, the net benefit between the nomogram and each independent risk factor for postoperative PVO was assessed by DCA. In this analysis, the nomogram provided a higher net benefit than all other factors, suggesting that the nomogram was superior in predicting the probability of postoperative PVO. As more patients are treated under the low-risk threshold, the net benefit tends to increase as the risk threshold is lowered. However, the low-risk threshold led to an increase in false-positive rates and unnecessary interventions. Next, clinical impact curves were generated to analyze the number of patients classified as high risk at each threshold and the number of patients classified as high risk. As shown in Figure 5, the difference between the total number of patients considered at high risk for postoperative PVO and the actual number of patients who failed is widening as the risk threshold decreases, which means an increase in false-positive rates and an increase in unnecessary treatment. Therefore, we adjusted DCA according to the clinical impact curve to strike a balance between higher net benefits and lower false-positive rates. The calibration results showed that when the risk threshold for postoperative PVO was set at 0.23, it provided the most significant clinical benefit for the entire included population, with the score falling just above the total score of 108 (Figures 3, 5).

### Comment

Since PVO with TAPVC repair is rare and highly individualized, it is extremely difficult to explore the risk factors of the disease (15, 16). All patients with TAPVC were incorporated into this study that includes those with pulmonary venous pressure and other heart disease patients. With the help of the logistic model and nomogram model of risk analysis, we found that the patients' preoperative pulmonary hypertension, surgery method, and preoperative pulmonary venous stenosis were associated with PVO independently (Table 3). Moreover, in terms of the prediction of risk, although preoperative pulmonary venous stenosis was one of the predictors of postoperative PVO, the preoperative pulmonary hypertension of patients showed a better predictive value than preoperative obstruction (Figure 2). In recent years, some experts have suggested that preoperative pulmonary venous stenosis and preoperative pulmonary hypertension are risk factors for PVO with TAPVC repair and have good predictive value (2, 17–20). However, it is worth noting that these studies analyzed the clinical value of the preoperative pulmonary venous stenosis in predictability PVO with TAPVC repair, but not quantitatively and weighted hierarchical analysis. Secondly, these studies showed us the risk factors associated with postoperative venous obstruction with TAPVC repair and provided qualitative conclusions, but did not elaborate on the predictive value of these risk factors. In other words, the prediction ability is proposed but the efficiency is not analyzed. It only relies on subjective experience to judge the predictive value, which is not rigorous enough, lacks a reliable basis, and is not objective enough in the conclusion.

**Figure 6:**
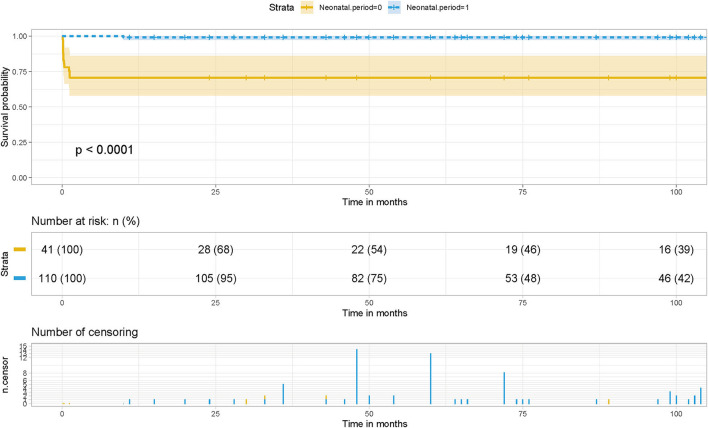
Effect of whether surgery during neonatal period on postoperative survival in patients with TAPVC and the cumulative risk (log-rank test). The x-axis and y-axis represent the postoperative follow-up time and survival probability, respectively [in yellow (neonatal period = 0, in neonatal period), the line represents the survival curve, and the width of the yellow bar represents the cumulative risk (the larger the width, the higher the cumulative risk). In blue (neonatal period = 1, out of the neonatal period), the line represents the survival curve, and the width of the blue bar represents the cumulative risk (the greater the width, the higher the cumulative risk.

It is worth noting that the earlier TAPVC symptoms appear, the more severe the condition is, the higher the proportion of patients with restrictive atrial septal defect, pulmonary vein stenosis, and severe pulmonary hypertension, the more emergency surgery is needed, and the greater the risk of surgery. Although there are serious physiological disorders and early hemodynamic instability in TAPVC, most newborns and infants can obtain good surgical results with active treatment and accurate diagnosis of preoperative and postoperative pulmonary hypertension. However, children often cannot get timely treatment in the neonatal period due to the family's low attention and insufficient cognition in the past times, as shown in Table 1, what is interesting is that we found whether treatment in the neonatal period was not a risk factor for postoperative pulmonary vein stenosis and obstruction after the chi-square test and Pearson test. While whether emergency treatment in the neonatal period had a significant difference in the survival status of children (*p* <0.0001) as shown in the survival curve (Figure 6). Therefore, it is reasonable to believe that the management of total anomalous pulmonary venous drainage in the neonatal period is not a risk factor for postoperative pulmonary vein stenosis, but it significantly affects the postoperative survival rate of children, and it still needs to be actively treated in the earlier stage.

**Figure 7:**
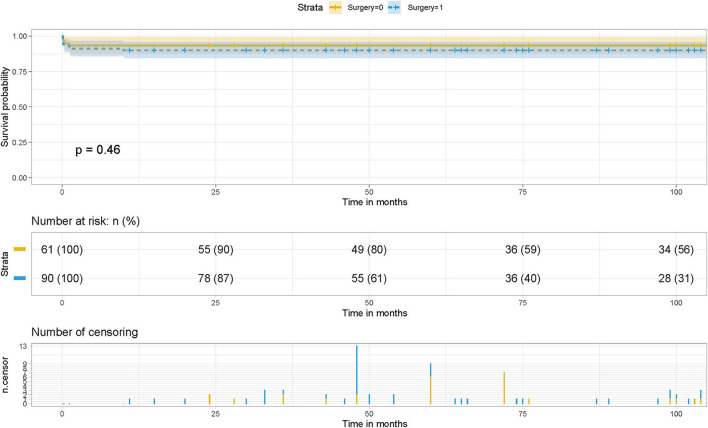
Effect of surgery on postoperative survival in patients with TAPVC and the cumulative risk (log-rank test). The x-axis and y-axis represent the postoperative follow-up time and survival probability, respectively [in yellow (Surgery = 1, non-sutureless surgery), the line represents the survival curve, and the width of the yellow bar represents the cumulative risk (the larger the width, the higher the cumulative risk). In blue (Surgery = 0, sutureless technique), the line represents the survival curve, and the width of the blue bar represents the cumulative risk (the greater the width, the higher the cumulative risk.

A recent meta-analysis of 26 studies involving a total of 2,702 patients showed that sutureless technology reduced the incidence of postoperative PVO and reoperation compared with routine surgery (20). Compared with routine surgery, the sutureless technique was associated with a lower occurrence rate of postoperative PVO and re-operations due to PVO. Meanwhile, postoperative early, late, and overall mortality were not statistically different between the two surgical approaches. The sutureless technique is beneficial in the primary repair of TAPVC regarding postoperative PVO and re-operations due to PVO (21, 22). This was consistent with our conclusions. Survival analysis suggested that although there was no statistical difference in the influence of the two surgical methods on the survival rate of patients, they had predictive value in the risk of postoperative PVO and the assessment of long-term survival of patients, and different surgical methods affected the incidence of postoperative PVO (Figure 7).

**Figure 8:**
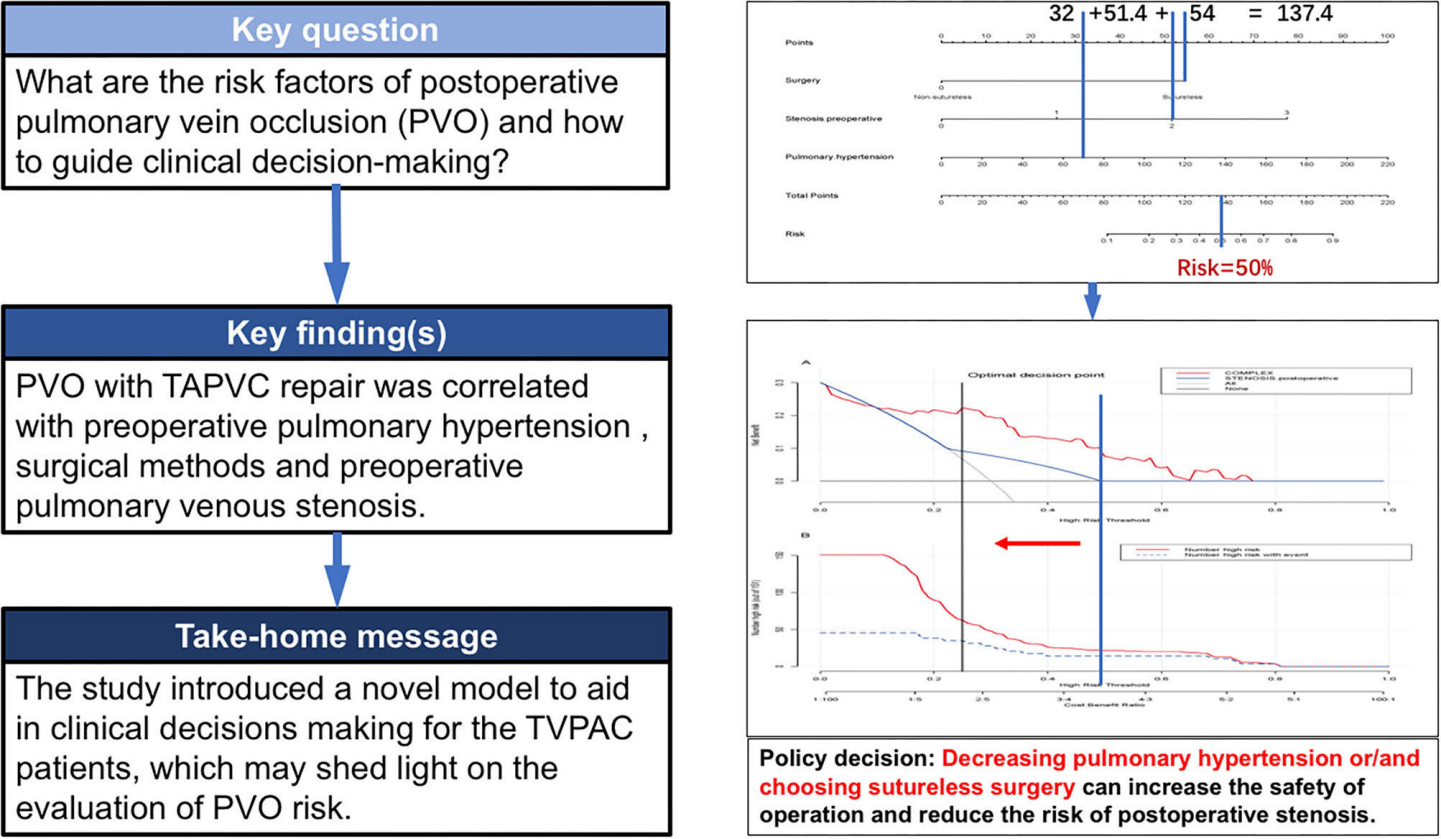
Clinical nomogram for Predicting the Prognosis of Patients with Pulmonary Venous Obstruction after Total anomalous pulmonary venous connection Repair.

This study establishes a nomogram model for the PVO risk and long-term survival prediction in patients with TAPVC repair based on preoperative pulmonary hypertension, surgical method, and preoperative pulmonary venous stenosis. The nomogram models make complex predictions more intuitive, easy to generalize, and useful for risk stratification and prognostic guidance in patients with this rare heart disease. In recent years, nomogram models have been widely used in oncology surgery to quantify disease risk by analyzing and considering all known clinical variables, thus allowing for individualized risk assessment and prognostic prediction for multiple cancers (8–11). Further combining the decision curve and the clinical impact curve, the optimal decision point is determined to achieve the optimization of the clinical decision by balancing the higher net benefit and the lower false-positive rate (Figure 8).

However, some limitations need to be acknowledged. First, this study was limited by retrospective analysis, so further studies are needed to verify this before expanding the clinical application of the nomogram. In addition, this study only included single-center samples, so it should be carefully checked and confirmed when applied in other populations and medical centers. Secondly, there are some changes in surgical technique and surgical proficiency due to the large period of this study, which make the results disturbed. Thirdly, although this is a large study, the sample size is still small, which hinders us from conducting a more meaningful subgroup analysis. In future studies, a more representative multicenter sample is needed to further refine the value of the risk assessment of the nomogram.

## Conclusions

In conclusion, the nomograph model established based on preoperative pulmonary hypertension, surgical method, and preoperative pulmonary venous stenosis realizes individual risk assessment and prognosis guidance for postoperative PVO) in patients with TAPVC repair and provides an objective and preliminary reference for clinical decision making and prognosis guidance.

## Data Availability Statement

The raw data supporting the conclusions of this article will be made available by the authors, without undue reservation.

## Ethics Statement

Written informed consent was obtained from the individual(s), and minor(s)' legal guardian/next of kin, for the publication of any potentially identifiable images or data included in this article.

## Author Contributions

LiaC and LinC designed the study and submitted the manuscript. LinC prepared the first draft of the manuscript and made the literature review. ZQ and FX made substantial changes in the manuscript together. XC collected and analyzed data together. All authors read and approved the final manuscript.

## Funding

This study received the financial support of the project for refractory diseases in cardio-macrovascular surgery. This paper was supported by Fujian Key Laboratory of Cardio-Thoracic Surgery (Fujian Medical University).

## Conflict of Interest

The authors declare that the research was conducted in the absence of any commercial or financial relationships that could be construed as a potential conflict of interest.

## Publisher's Note

All claims expressed in this article are solely those of the authors and do not necessarily represent those of their affiliated organizations, or those of the publisher, the editors and the reviewers. Any product that may be evaluated in this article, or claim that may be made by its manufacturer, is not guaranteed or endorsed by the publisher.
